# Supplementary material for: The cGAS/STING/IFN-1 Response in Squamous Head and Neck Cancer Cells after Genotoxic Challenges and Abrogation of the ATR-Chk1 and Fanconi Anemia Axis
Source: Int J Mol Sci. 2023 Oct 4;24(19):14900. doi: 10.3390/ijms241914900 (PMC10573837; doi:10.3390/ijms241914900)

Supplementary Figure S1:

Quantitative measurement of protein expression based on the integrated optical intensity of chemiluminescence signals of Western blots shown in (A) Fig. 4A and (B) Fig. 4B performed as previously described in [64]. Values obtained are shown as relative protein levels after normalization to the loading control vinculin and to the level of mock-treated cells (solid line).

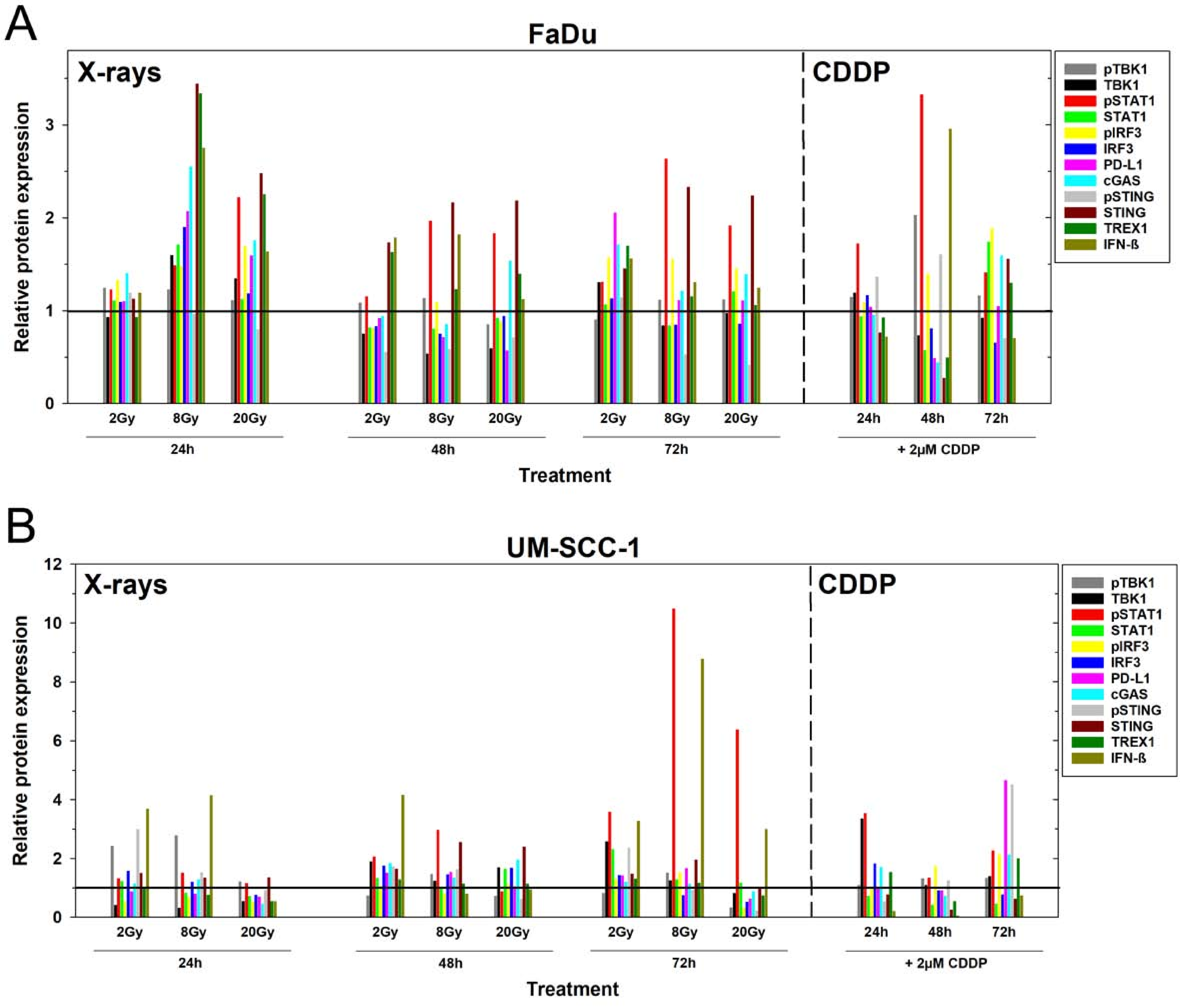

Supplementary Figure S2:
Western blots of (A and C) FaDu and (E and G) UM-SCC-1 cells after exposure to 0, 2, 8, or 20 Gy X-rays or 2 µM Cisplatin (CDDP) without or with inhibition of ATR, Chk1, or Fanconi anemia gene A knockout (FANCA ko) 72 h after the start of treatments as exemplarily shown in Fig. 4C and D. Western blots show the three independent replicate experiments for each HNSCC cell line. Quantitative measurement of protein expression based on the integrated optical intensity of chemiluminescence signals is shown in B, D, G and H and was performed as previously described in [64]. Values obtained are shown as relative protein levels after normalization to the loading control vinculin and to the level of (B and G) mock-treated cells without exposure to X-rays or CDDP or (G and H) without abrogation of the DNA damage response (Ø) (solid line). Data are expressed as mean and standard deviation.

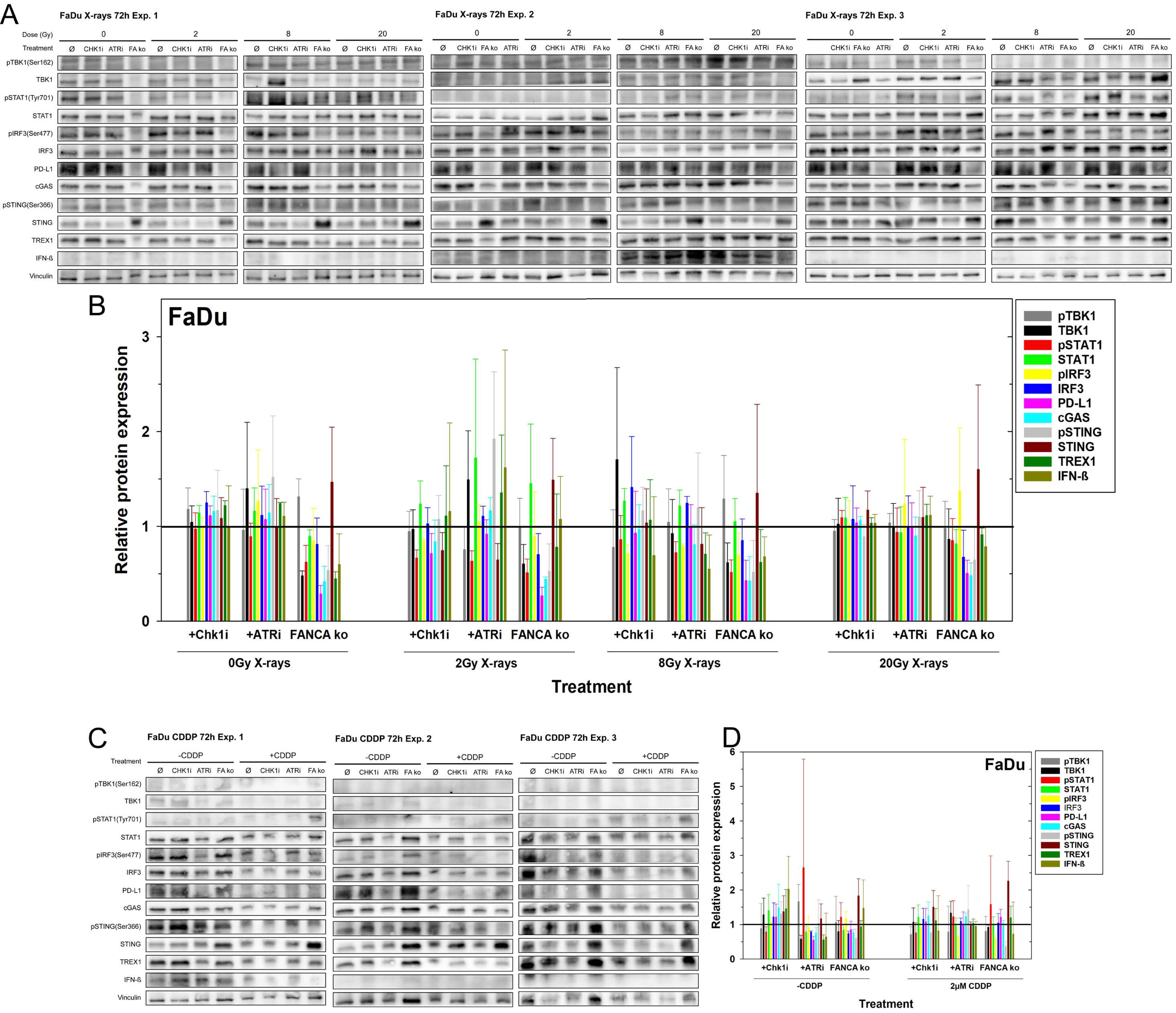

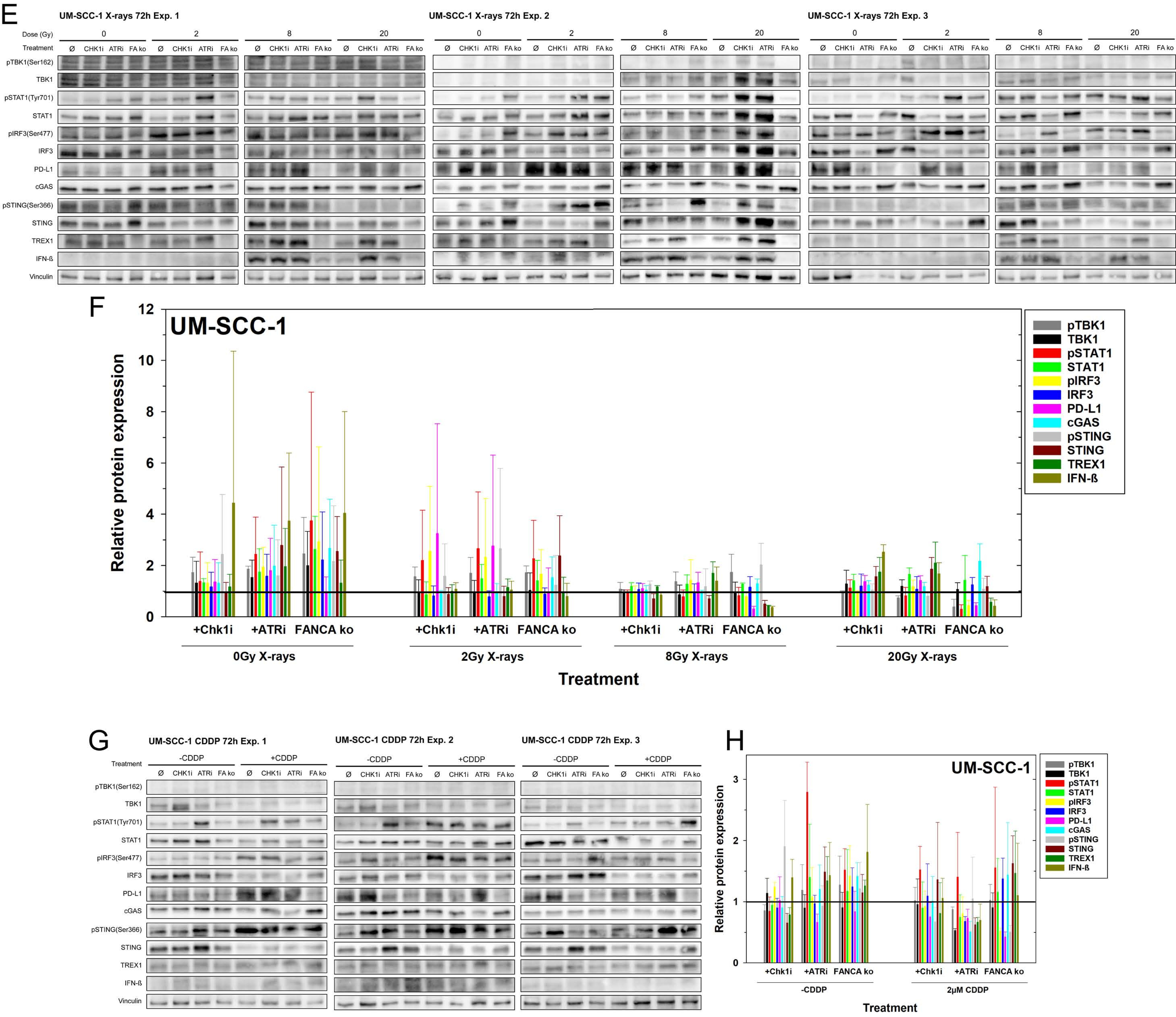
E
UM-SCC-1 X-rays 72h Exp. 1
UM-SCC-1 X-rays 72h Exp. 2
UM-SCC-1 X-rays 72h Exp. 3
Dose (Gy)
0
2
8
20
Treatment
Ø
CHK1i
ATRi
FA ko
pTBK1(Ser162)
TBK1
pSTAT1(Tyr701)
STAT1
pIRF3(Ser477)
IRF3
PD-L1
cGAS
pSTING(Ser366)
STING
TREX1
IFN-ß
Vinculin
F
UM-SCC-1
Relative protein expression
pTBK1
TBK1
pSTAT1
STAT1
pIRF3
IRF3
PD-L1
cGAS
pSTING
STING
TREX1
IFN-ß
+Chk1i
+ATRi
FANCA ko
0Gy X-rays
2Gy X-rays
8Gy X-rays
20Gy X-rays
Treatment
G
UM-SCC-1 CDDP 72h Exp. 1
UM-SCC-1 CDDP 72h Exp. 2
UM-SCC-1 CDDP 72h Exp. 3
-CDDP
+CDDP
H
UM-SCC-1
-CDDP
2µM CDDP
Treatment

Supplementary Figure S3:

(A) Western blots analysis of protein levels of TBK1, STAT1, IRF3, cGAS, STING, and ß-actin in FaDu (hypopharyngeal), UM-SCC-1 (oral cavity/ mouth base), SCC-9 (tongue), Cal-33 (tongue), SCC-25 (tongue), UPCI-SCC-131 (oral cavity/ mouth base), SCC-263 (oral cavity/ mouth base) HNSCC cell lines and WI-38 and MRC5 primary human lung fibroblasts. The cell lysates were generated in previous studies and further information can be found therein [43, 64].

(B) The bar chart shows the corresponding expression levels of the target proteins relative to ß-actin (solid line) for each cell line.

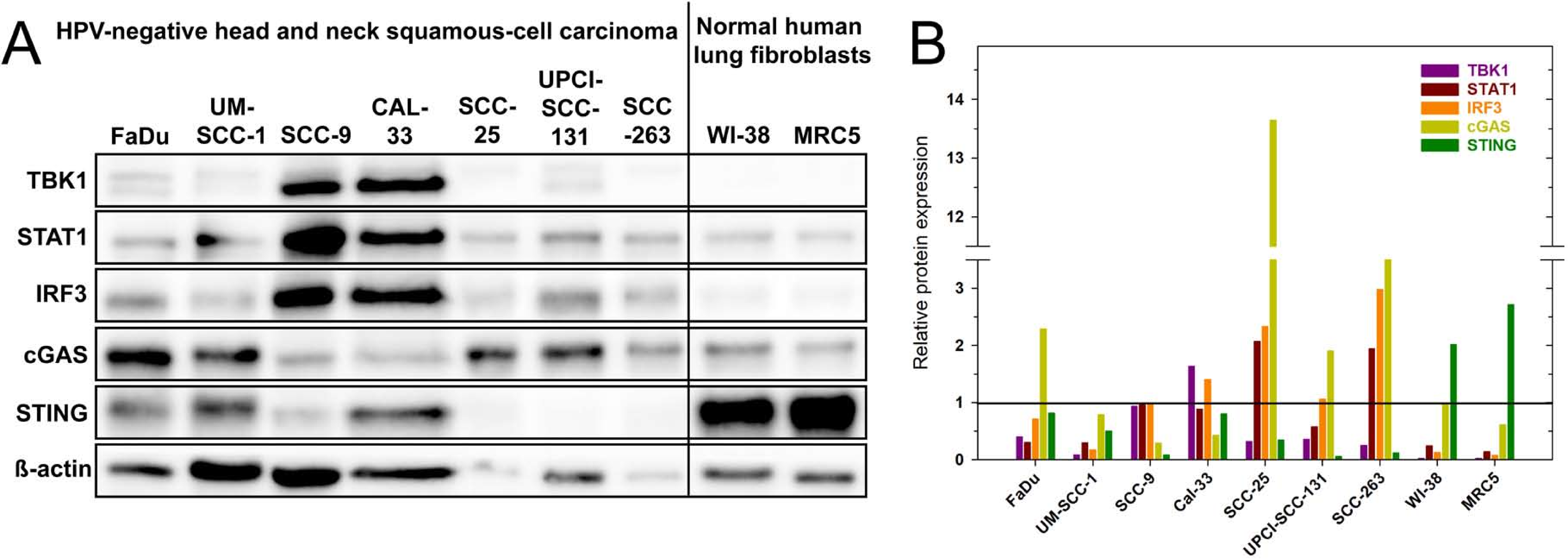

Supplementary Figure S4:

The FANCA ko in FaDu (A) and UM-SCC-1 (B) cells was confirmed by cell cycle analysis after treatment with the interstrand crosslink inductor mitomycin C (MMC, medac, Germany). Exponentially growing cells were treated for 24h with MMC, fixed, stained, and analyzed by flow cytometry as described previously (40). Compared to HNSCC wt cells, FANCA ko induced a strong G2/M arrest after MMC treatment.

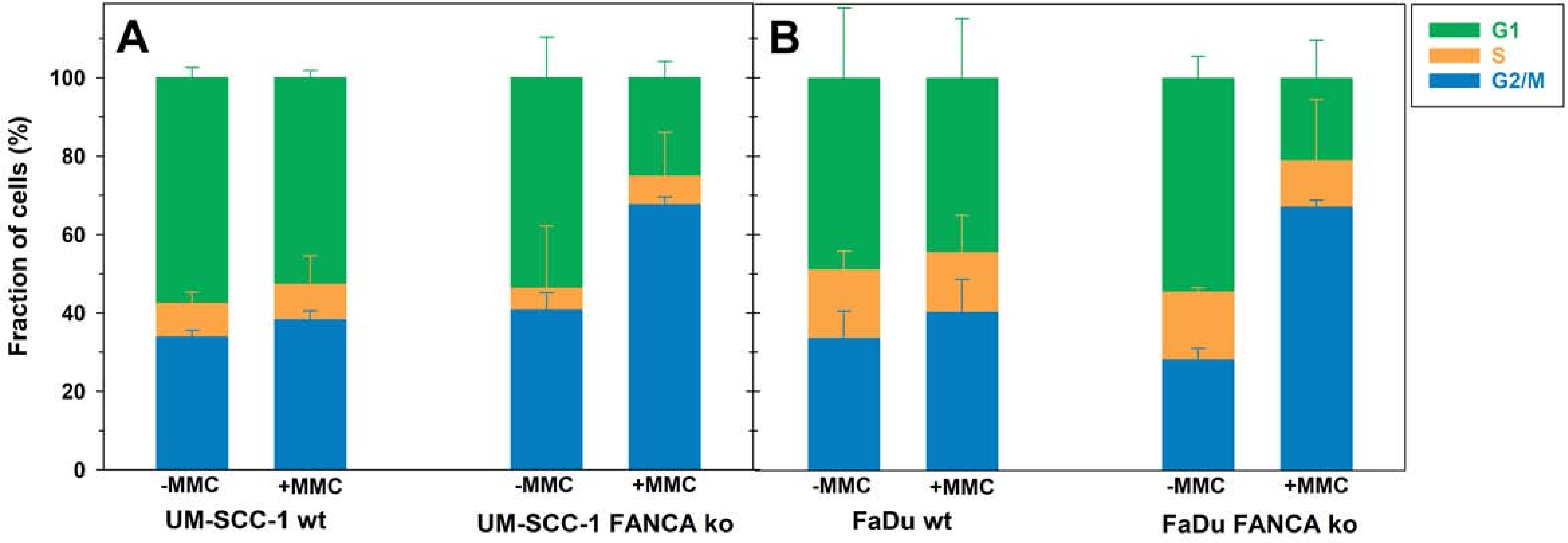

Supplementary Figure S5:
Reference curve for the quantification of cytosolic double-stranded DNA with the Pico488 dsDNA quantification kit (Lumiprobe, Germany) according to the manufacturer's protocol. Data were pooled from all measurements with FaDu and UM-SCC-1 cells (n=12). A linear fit was applied.

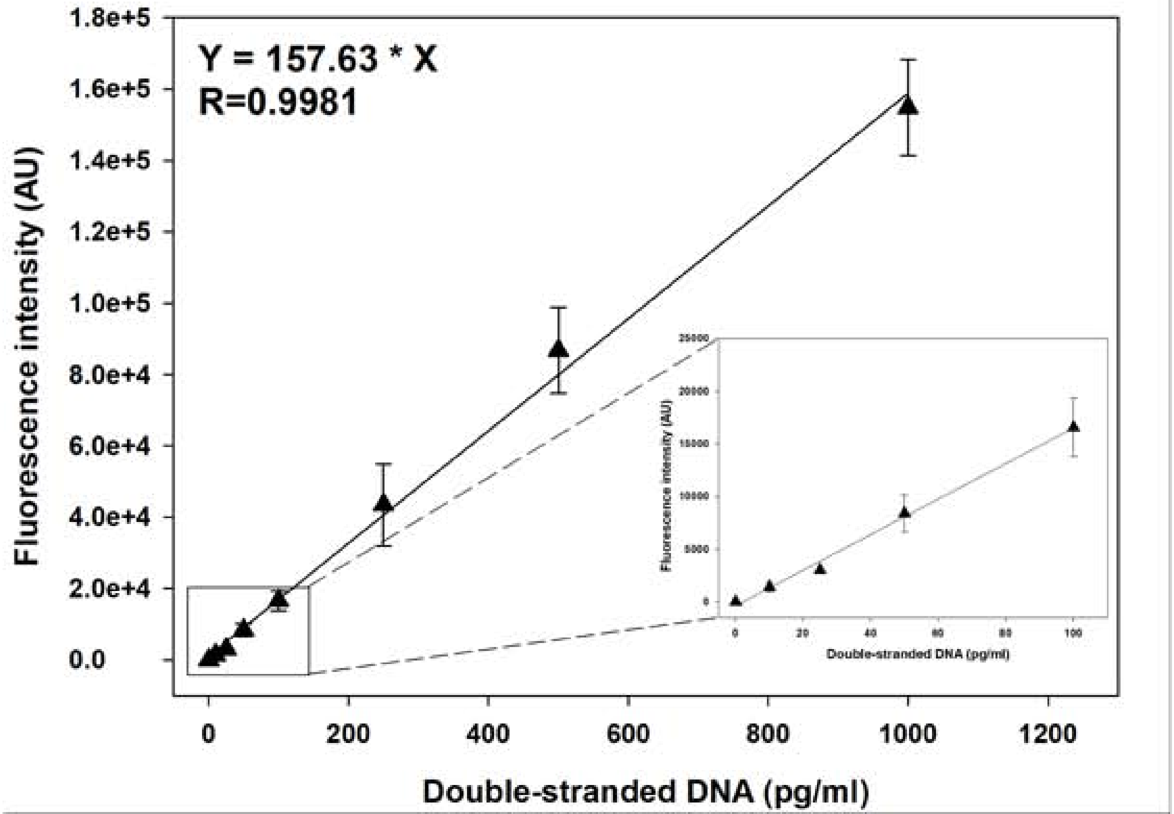

Supplement: Supplementary file 1 [file ijms-24-14900-s001.zip › ijms-2632792-supplementary.pdf]
